# Influence of different skew angles of the submerged vane on pressure flushing performance

**DOI:** 10.1038/s41598-023-41276-1

**Published:** 2023-08-30

**Authors:** Sepideh Beiramipour, Sameh A. Kantoush, Hadi Haghjouei, Kourosh Qaderi, Majid Rahimpour

**Affiliations:** 1 Department of Water Engineering, Shahid Bahonar University of Kerman, Kerman, Iran; 2 Disaster Prevention Research Institute, Kyoto University, Kyoto, Japan

**Keywords:** Civil engineering, Hydrology

## Abstract

Siltation significantly threatens a reservoir’s original storage capacity and lifespan. Pressure flushing is an effective measure against siltation through the partial drawdown of the reservoir water level with limited flushed cone volumes in front of the bottom outlet. In this study, a novel configuration with submerged vanes has been proposed and tested experimentally to increase the flushed sediment volume during pressure flushing. In the new configuration, submerged vanes aligned with ten skew angles (θ) of 10°, 20°, 30°, 45°, 70°, 110°, 135°, 150°, 160°, and 170° to the flow direction in noncohesive sediment bed materials were used. The results showed that increasing the skew angle at 45° ≤ θ ≤ 160° increased the flushing cone geometry. The minimum and maximum flushing cone dimensions and volume occurred at skew angles of θ = 45° and 135° ≤ θ ≤ 160° around the bottom outlet. The sediment flushing volume in the presence of submerged vanes at 135° ≤ θ ≤ 160° increased 25 times compared to tests without submerged vanes. Eventually, nonlinear regression analysis yielded an equation for estimating flushing cone volumes. The developed equation was tested in real case studies of a target reservoir, and an acceptable correlation between the calculated and experimental results was obtained.

## Introduction

When a river approaches a reservoir, the flow velocity decreases due to changes in the cross-sectional area, which reduces the transport capacity of flow and finally leads to sedimentation in the reservoir. Therefore, sediments accumulate behind the dam and occupy a larger reservoir volume over time. As a result, sedimentation steadily reduces reservoir capacity worldwide, threatening the reliability of water supplies, flood control, hydropower energy, and other benefits that form the basis of today’s water-intensive society^1^.

Reservoir function cannot be sustained without implementing sediment management strategies^2^. Management strategies may be classified into four categories: (a) reducing sediment yield from the watershed, (b) routing sediment-laden flows around or through the storage pool, (c) removing sediment following deposition, and (d) adaptive strategies that respond to capacity loss^1^. Among these strategies, hydraulic flushing is an effective method for evacuating deposited sediments from reservoirs by opening bottom outlets. This method can be classified as free surface and pressure flushing^3^. Pressure flushing, the partial water level drawdown, is operationally affordable, especially in arid and semiarid regions. Additionally, pressure flushing is essential for hydropower dams in arid and semiarid countries where over-year storage is necessary. In pressure flushing, the water level is sustained in the reservoir, and deposited sediment can be scoured rapidly near the sluice gate opening. Additionally, pressure flushing can reduce clogging in front of the bottom outlet and recover a fraction of reservoir capacity by scouring a small amount of sediment in the region near the bottom outlet. In this method, a cavity or hollow like a cone or funnel develops in front of the bottom outlet^4^.

Three main disadvantages of pressure flushing are the limited ability to recover a large amount of storage capacity, low efficiency, and effects only close to the low-level outlet in front of the dam. Therefore, this method should be combined with structural techniques for improvement. Many auxiliary structures have been investigated to increase the performance of pressure flushing. Most of these works intend to enhance the sediment removal capacity by adding accessory structures to traditional bottom outlets. The enhanced sediment removal capacity is due to the changes these new structures generate in the flow field around them compared to the flow field around traditional bottom outlets (orifice flow).

The mechanisms that enhance sediment removal efficiency in these works are different. For example, Ahadpour Dodaran et al.^5^ used vibrator plates to increase the volume and dimensions of flushing cones. Althaus et al.^6^ also investigated the application of water jets in flushing. In both studies, the auxiliary structures used electrical energy for sediment flushing operations, which increased costs. For energy savings, some studies were conducted by new facilities to enhance the strength of vortices. For instance, Haghjouei et al.^7–9^ proposed the DBE (Dendritic Bottomless Extended) structure to investigate the structural effects on the sediment flushing cone dimensions and efficiency due to vortices arising from the pressure difference between the inside and outside of the structure with the ability to flush at different sides of the reservoir. One of the most significant disadvantages of PBC and DBE is the problematic installation and operation of these structures and less amount of flushed sediment compared to these problems. For a more straightforward procedure and to enhance the efficiency of pressure flushing compared to previous studies, Beiramipour et al.^10,11^ used submerged vanes with a new divergent arrangement.

Additionally, in another study, Beiramipour^12^ improved the operation of the DBE structure by combining it with submerged vanes. In previous studies on the effect of submerged vanes in pressure flushing conducted by Beiramipour et al.^10^, more focus was placed on the performance of the vanes and their arrangements and the selection of the best geometric parameters of vanes. In this study, submerged vanes with different complementary skew angles aligned with the flow direction in other hydraulic conditions were used to increase the performance of hydraulic pressure flushing in reservoirs, and the best angles were proposed.

### Application of submerged vanes

Submerged vanes are small flow-training structures (foils) designed to modify near-bed flow patterns and redistribute flows and sediment transport within the channel cross-section. The structures are typically installed at an angle of attack of 10 to 20° relative to the flow, and high- and low-pressure zones are produced on both sides of the vanes. Their initial height is 0.2 to 0.4 times the local water depth at the design stage. The standard ratio of height to length of vanes is 0.3, and the ratio of vane height above the sediment bed to water level is recommended to be between 0.2 and 0.5^13,14^. The vanes function by generating secondary circulation in the flow. The circulation alters the magnitude and direction of the bed shear stresses and causes a change in the distributions of velocity, depth, and sediment transport in the area affected by the vanes. As a result, the riverbed degrades in one portion of the channel cross-section and degrades in another.

Generally, submerged vanes are used for river channel stabilization, bank protection, shoaling amelioration, control and reduction of scour, and sedimentation at diversions and water intakes^13–20^. Additionally, submerged vanes can be used to increase the turbulence of flows upstream of the bottom outlets of reservoirs and, finally, to increase the amount of evacuated sediment. By employing submerged vanes in reservoirs, vertical pressure gradients cause the formation of vortexes and rotational flows around the vanes. These rotational flows cause changes in bed shear stress and topography^21^. Due to the creation of resistant forces and eddy currents, the center of which is approximately at the level of 0.9 of the height of the vanes, sediment around the submerged vanes is gradually flushed, and by creating a horseshoe vortex around the vanes, local scouring is intensified, and a sediment flushing cone is formed^22^. Notably, the flow inside open channels differs in velocity and pressure profiles from the reservoir. The flow velocity inside the reservoir is low, and the turbulence conditions will occur only in the area near the bottom outlet.

Additionally, even though secondary currents form in a channel, the bulk of the flow moves in the direction of the flow approximately uniformly. Regardless of the vanes, the flow upstream of the bottom outlet cannot be considered uniform; it is a radial flow toward the orifice. Therefore, the direction and intensity of the turbulent structures generated by the vanes may differ from what is known about them in straight sections and bends of channels. Among the different design parameters of submerged vanes, the angle of attack of the vane with the flow is an important parameter, and it has been the subject of various investigations. For example, many researchers have studied the effect of the vane angle of attack on controlling and reducing scour^23–39^.

The results drawn from the above studies clarify that submerged vanes with different angles of attack and straight flow directions have been used to reduce scouring and sediment transport in open channels. Therefore, this study aimed to investigate the performance of submerged vanes aligned with skew angles of θ = 10°, 20°, 30°, 45°, 70°, 110°, 135°, 150°, 160°, and 170° to enhance the pressure flushing processes and efficiency. Furthermore, a nondimensional formula based on nonlinear regression analysis was developed to predict the pressure scour cone volumes. Tests were conducted without submerged vanes, and the results were compared with each other to evaluate the performance of submerged vanes.

## Materials and methods

### Materials

**Figure 1:**
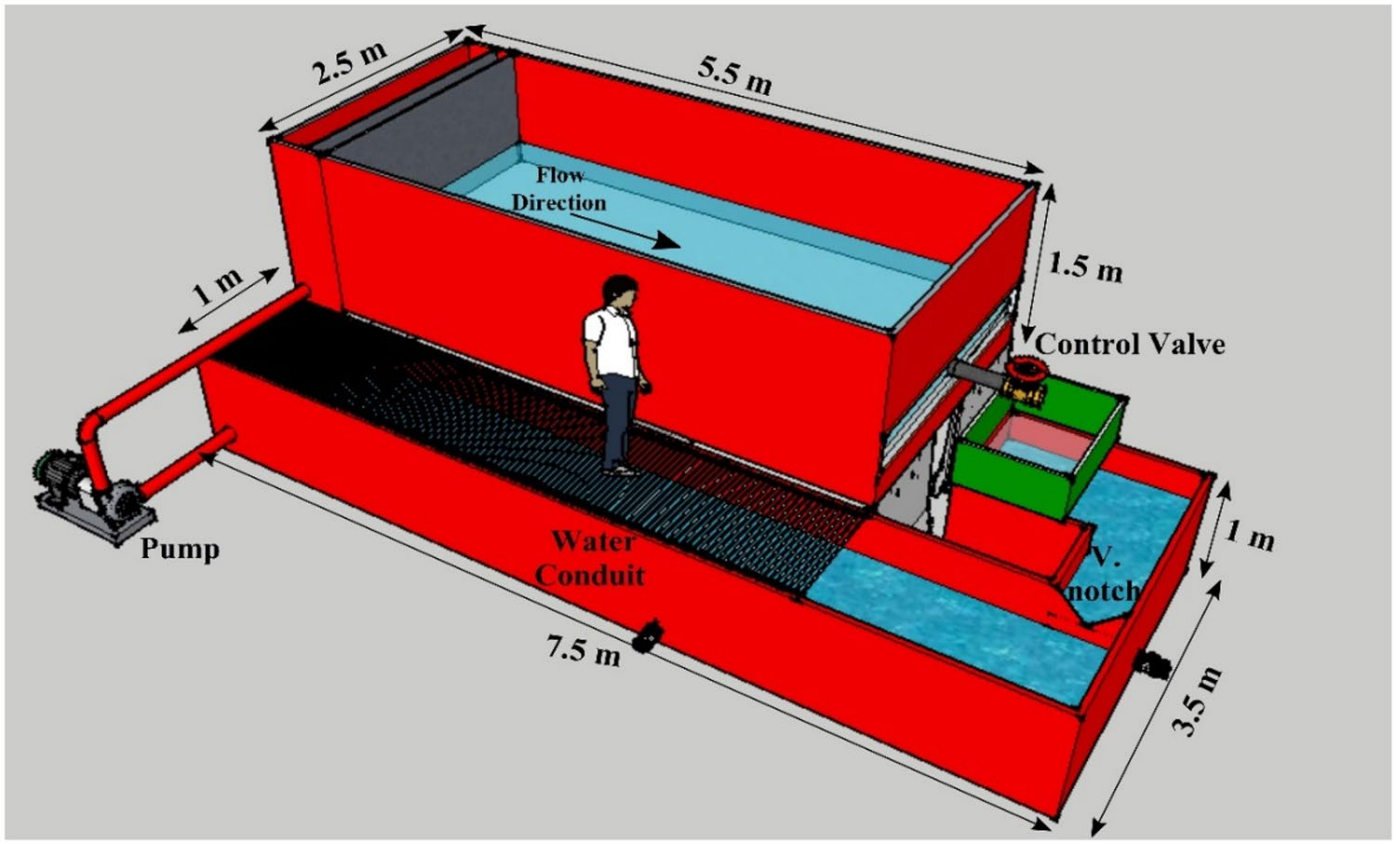
Schematic rrepresentation of the physical model.

Laboratory experiments were performed in a physical model of a dam 7.5 m in length, 3.5 m in width, and 1.8 m in height at the Hydraulic and Water Structures Research Laboratory of the Water Engineering Department of the Shahid Bahonar University of Kerman. This model was not based on specific existing reservoirs and prototypes but is instead an exhibition of the deposited sediment problem behind any dam reservoir. The bottom outlet had a circular shape with a diameter of 10 cm, and a control valve was placed in the middle of the front wall of the reservoir, which was made of glass to allow better observations of sediment transport. A water conduit and a centrifugal pump were used to fill the reservoir. The outflow from the reservoir was returned to the water conduit and recirculated through this system. The flow discharge was measured with a V-notch weir located at the first part of the water conduit. The schematic view of this model is shown in Fig. 1.

All tests were conducted with 12.5, 15, and 18 L s^−1^ outflow discharge rates. The water head and sediment level were 50 and 40 cm at the reservoir. The sediment used in the experiments consisted of noncohesive silica particles with a median diameter of d_50_ = 0.73 mm and a relative density of 2.65. Additionally, the geometric standard deviation of sediment (σ) was determined to be 1.49, which indicates a uniform size distribution because σ is less than 1.5^17,40^. To assess the optimal angle of the submerged vanes, galvanized plates were positioned (1) at different skew angles, (2) at different minimum spacing of vanes, and (3) with different outflow discharge rates.

### Experimental procedures

**Figure 2:**
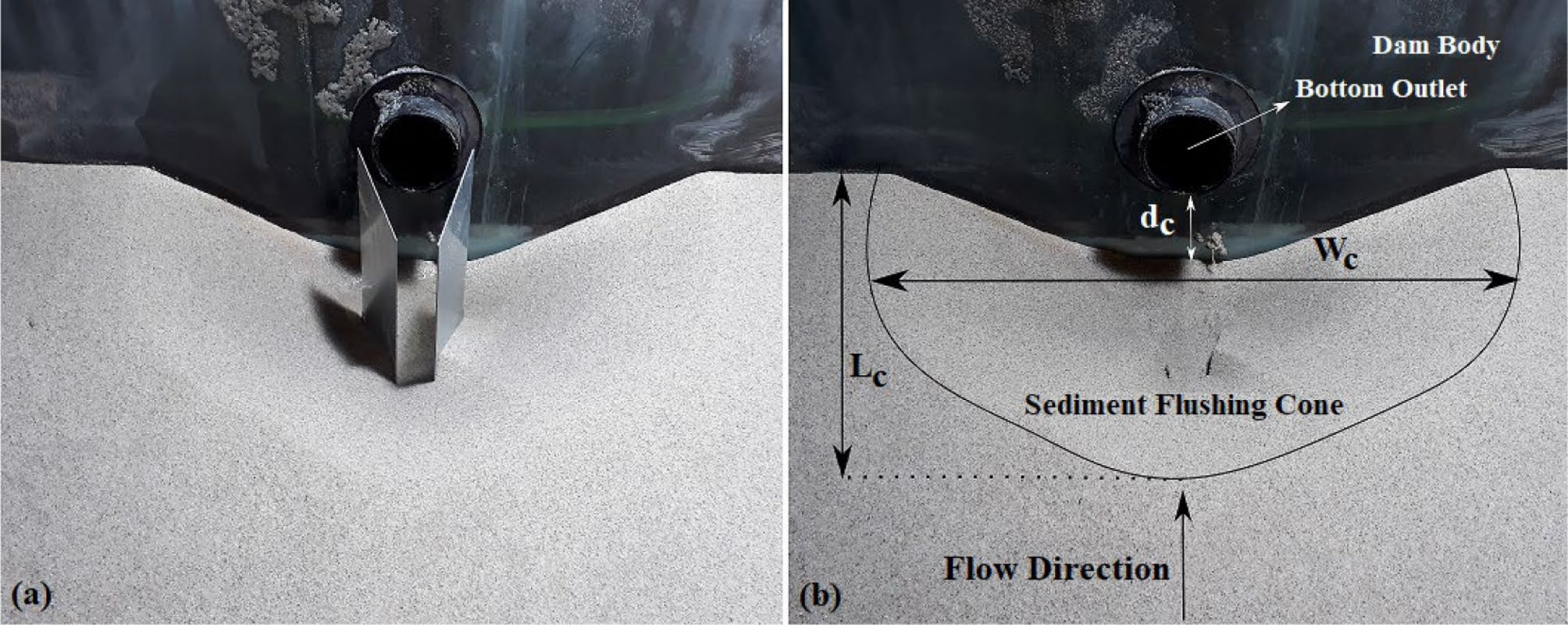
(**a**) A finished test with the presence of submerged vanes, (**b**) dimensions of the sediment flushing cone after removing the vanes.

Before the start of each test, the surface of the sediment bed in the reservoir was smoothed, and according to the designed scenarios, vanes were placed in the sediment. The reservoir was initially filled slowly to prevent disturbances to the surface of the packed sediment bed. Then, the centrifugal pump was turned on, and the discharge was set with a volumetric meter. The outlet valve opened when the water reached the desired level, and the test started. After the system reached dynamic equilibrium, the centrifugal pump was turned off, and the output valve was closed; water was drained with a valve located at the bottom of the reservoir. Then, the submerged vanes were removed very slowly from the sediment to measure the dimensions of the flushing cone and final analysis. It should be mentioned that a 12-h test established the temporal variations in the sediment flushing cone, and the equilibrium time was obtained. After three hours, more than 96% of the flushing cone was shaped, and after approximately six hours, the variation rate of the flushing cone was decreased, and the final scour cone was formed. After conducting each laboratory test, the geometry of the pressure flushing cones, including the maximum length (L_c_), maximum width (W_c_), and maximum depth (d_c_), were measured. The geometry of the scour cone that forms during pressure flushing is specifically essential. This is because the flushing event aims to remove accumulated sediment around the entrance of an intake and prevent the abrasion of hydraulic structures^41^. Figure 2a and b show a finished test before removing the submerged vanes and dimensions of the flushing cone after a test has been finished and submerged vanes were removed, respectively.

When the scour cone measurements were finalized, the acquired data were processed. To determine the volume of the flushing cone, a Canon IXUS 190 camera with remote control was used, the photos were analyzed by a photo scanning technique (PST), and point cloud files were converted, aligned, and analyzed^42^. The point cloud file was oriented to the axis system using the aligning tools of the Agisoft software^43^. After that, a surface object was made from the point cloud, and the points were extracted from the surface. Finally, all points were imported into ArcGIS^44^, and the volume calculations were performed by taking the volumetric difference between the original sediment surface and the surface of the scour cone. Using the XYZ data exported from ArcGIS, the topography of each scour cone was prepared in Surfer-16 software^45^. The profile tool in Surfer exported longitudinal and transverse profile data to Microsoft Excel, where they were processed and plotted. It is noticeable that the PST was calibrated on a cube with known dimensions and volume. The data accuracy at a distance of 50 cm was determined to be 0.35%, which equates to an error of approximately 1.75 mm.

### Dimensional analysis

**Figure 3:**
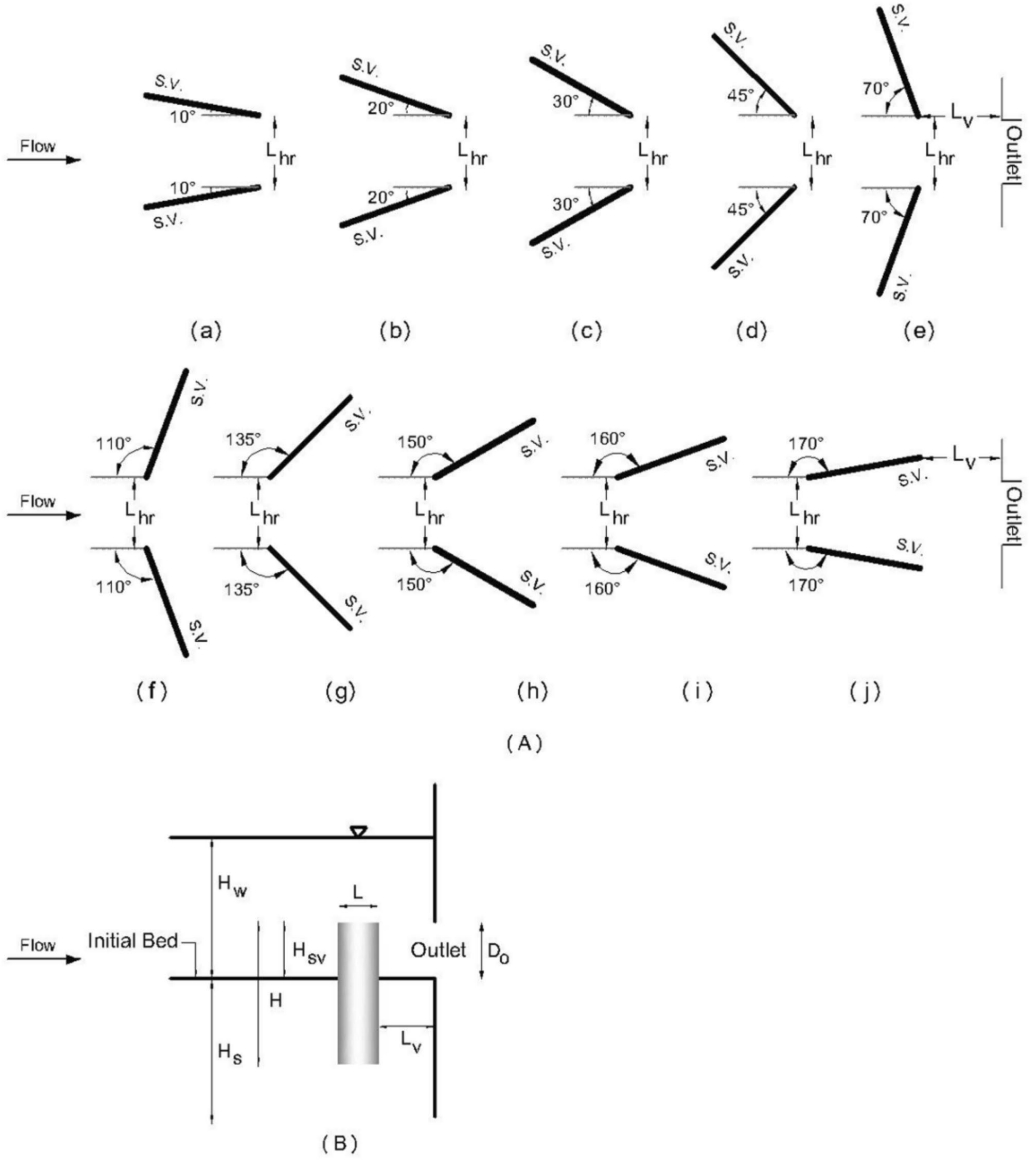
Schema of (**A**) plan view of vanes at (**a**) θ = 10°, (**b**) θ = 20°, (**c**) θ = 30°, (**d**) θ = 45°, (**e**) θ = 70°, (**f**) θ = 110°, (**g**) θ = 135°, (**h**) θ = 150°, (**i**) θ = 160° and (**j**) θ = 170° and (**B**) longitudinal view of vanes with geometric parameters.

The maximum volume of the sediment flushing cone ($V_{c}$) around submerged vanes with a skew angle of $\theta$ concerning the flow direction depends on the hydraulic and sediment characteristics and geometric dimensions of the submerged vanes and outlet and can be described by the following functional relationship:

1 $$ V_{c} = f_{1} \left( {v_{o} , H_{w} , H_{s} ,d_{50} , \rho_{w} , \rho_{s} , \mu , g, D_{o} , H_{sv} , L_{sv} , L_{hr} ,L_{v} , \theta } \right) $$

where $f_{1}$ = an unknown function, $v_{o}$ = flow velocity at the bottom outlet, *H*_*w*_ = the total water head, *H*_*s*_ = the sediment level, $d_{50}$ = the median size of the sediment particles, $\rho_{w}$ = the fluid density, $\rho_{s}$ = the sediment density, $\mu$ = the fluid viscosity, $g$ = acceleration due to gravity, $D_{o}$ = the diameter of the bottom outlet, $H_{sv}$ = the height of the submerged vanes above the sediment bed, $L_{sv}$ = the length of the submerged vanes, $L_{hr}$ = the minimum spacing of submerged vanes, $L_{v}$ = the distance of the submerged vanes from the outlet, and *θ* = the angle of the submerged vanes for the straight flow direction. It should be noted that the sediment level (*H*_*s*_) corresponded with the sill level of the outlet, and the other parameters (*H*_*w*_ and ${\text{H}}_{{{\text{sv}}}}$) were measured at this level. Figure 3 schematically shows the alignment of the vanes with ten different skew angles and geometric parameters.

By selecting $v_{o}$, $\rho_{w}$ and $D_{o}$ as the repeating variables and according to the Buckingham theorem by substituting $\frac{{\rho_{s} }}{{\rho_{w} }}$ as the sediment-specific gravity ($G_{s}$), $\frac{{\rho_{w} v_{o} D_{o} }}{\mu }$ as the Reynolds number (Re) and $\frac{{v_{o} }}{{\sqrt {g D_{o} } }}$ as the Froude number (Fr) in Eq. (1), the dimensionless terms can be represented as follows:

2 $$ \frac{{V_{c} }}{{D_{o}^{3} }} = f_{2} \left( {{\text{Fr}}, \frac{{H_{w} }}{{D_{o} }},\frac{{H_{s} }}{{D_{o} }}, \frac{{d_{50} }}{{D_{o} }}, G_{s} , {\text{Re}}, \frac{{H_{sv} }}{{D_{o} }}, \frac{{L_{sv} }}{{D_{o} }}, \frac{{L_{hr} }}{{D_{o} }}, \frac{{L_{v} }}{{D_{o} }}, \theta } \right) $$

In this study, the sediment-specific gravity ($$G_{s}$$) was considered constant because the same sediment material (only noncohesive silica) was used, and $$G_{s}$$ was determined to be $$ 2.625$$. The Reynolds number (Re) was considered negligible under a fully turbulent flow from the bottom outlet. Additionally, in all experiments, vanes with the same length were used. Therefore, $$\frac{{H_{w} }}{{D_{o} }}$$, $$\frac{{H_{s} }}{{D_{o} }}, \frac{{d_{50} }}{{D_{o} }},{\text{ and }}\frac{{L_{sv} }}{{D_{o} }}$$ remained constant.

The height of the submerged vanes above the sediment bed ($$H_{sv} )$$ and the distance of the submerged vanes from the outlet ($$L_{v} )$$ were considered according to the results of Beiramipour et al.^10,11^. They investigated the effects of vane distances from the outlet and vane heights above the sediment bed. They observed that the reduction of $$L_{v}$$ improved flushing performance and suggested $$\frac{{L_{v} }}{{D_{o} }}$$ = 0.3. Furthermore, by analyzing the different heights of vanes with various arrangements, they suggested that for convergent arrangements of submerged vanes (θ = 10°, 20°, 30°, 45° and 70° in this study), H_sv_/D_o_ should be 2, and for divergent arrangements of submerged vanes (θ = 110°, 135°, 150°, 160° and 170° in this study), H_sv_/D_o_ should be 1. It should be mentioned that according to the standard ratio of vanes height above the sediment bed to water level, this parameter was determined to be 0.2 and 0.4 in this study. Thus, Eq. (2) can be simplified as follows:

3 $$ \frac{{V_{c} }}{{D_{o}^{3} }} = f_{2} \left( {{\text{Fr}}, \frac{{L_{hr} }}{{D_{o} }},\theta } \right) $$

According to Eq. (3), the effect of the dimensionless variation in Fr, L_hr_/D_o,_ and θ on the pressure flushing performance is investigated in this research. Table 1 presents the experimental conditions and dimensionless parameters.

**Table 1:** Description of the experiments.

Test category	*D*_*o*_ (cm)	*Fr*_*o*_	$\theta$(°)	*L*_*hr*_ (cm)	*L*_*v*_ (cm)	*H*_*sv*_ (cm)	$\frac{{L_{hr} }}{{D_{o} }}$	$\frac{{L_{v} }}{{D_{o} }}$	$\frac{{H_{sv} }}{{D_{o} }}$	Number of tests
Without vanes (reference tests)	10	1.6, 1.92, 2.32	–	–	–	–	–	–	–	3
With vanes	10	1.6, 1.92, 2.32	10, 20, 30, 45, 70	5, 10, 15	3	20	0.5, 1, 1.5	0.3	2	45
With vanes	10	1.6, 1.92, 2.32	110, 135, 150, 160, 170	5, 10, 15	3	10	0.5, 1, 1.5	0.3	1	45

## Results and discussion

### Experimental observations

The main reason for flushing is to evacuate deposited sediment from the reservoir. The pressure flushing through bottom outlets acts by two factors: the bottom outlets' local effect and the reservoir's general flow movement. In reference tests, only the local effect of the bottom outlets erodes sediment from the reservoir. This region, near the bottom outlet, is minimal and cannot contribute to sediment flushing from the bulk of the reservoir. In the reference tests in the current study, as the bottom outlet was opened, sediment scoured out close to the outlet. This scour at the opening formed quickly after water flowing through the outlet was clear. These observations are consistent with the observations of White and Bettess^46^, Di Silvio^47^, Powell and Khan^48^, and Kondolf et al.^49^.

However, this region extended in the presence of submerged vanes with different skew angles, and sediment was evacuated with high intensity. Based on the features of flow around submerged vanes observed from video recording combined with some described by Odgaard and Kennedy^13^, Odgaard and Wang^31^, and Odgaard^14^ and the erosion pattern we observed in our experiments, it was concluded that the flow downstream of the vane with high turbulence formed a pair of clockwise and counterclockwise vortices. The circulating flows interacted with the secondary currents generated by the vanes, and rotational flow formed; thus, the sediments rotated with severe vortices. In this way, a groove was created between the bottom outlet and vanes, which directed the transfer of part of the sediments from upstream to downstream. Finally, deposited sediments collected in front of the bottom outlet were evacuated. In this study, the flow behavior around submerged vanes as a turbulent structure was similar to the findings of previous studies that used submerged vanes in rivers.

**Figure 4:**
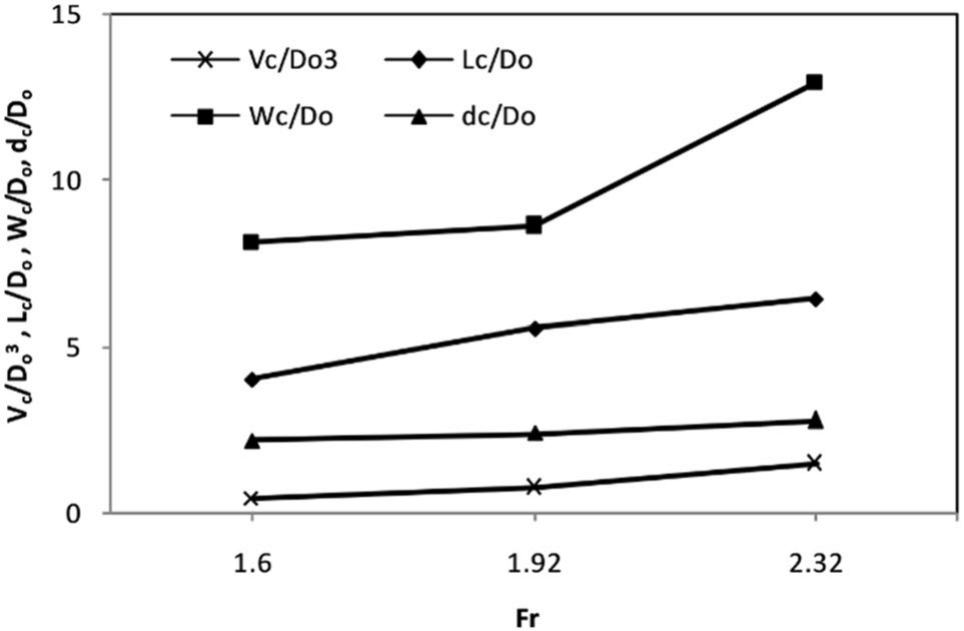
Variations in the volumes and dimensions of the sediment flushing cones with increasing Froude number in reference tests.

Outflow discharge is an essential property of pressure flushing. In this study, the tests were carried out with different outflow discharges $({\text{Q}}_{{\text{o}}} )$ of 12.5, 15, and 18 L s^−1^. Figure 4 shows increased scour cone size with increasing discharge, and a Froude number was obtained. The increase in discharge through bottom outlets to flush sediments strengthens the pressure gradient and increases sediment removal. The vertical gradients of velocity and bed shear stress reinforce a pulling effect on sediment particles. Thus, a higher flushing rate and more giant cones are produced^50^. This is consistent with the observations of several researchers^4,40,51^. For the range of outflow discharge in this study, an average increase of 1.44 times in discharge causes the flushing cone volume, length, width, and depth to increase by approximately 3.4, 1.6, 1.6, and 1.3 times, respectively.

### The effect of skew angles of vanes on their performance

#### for skew angles less than 45°

To investigate the effects of different vane angles, the angles are classified into two groups: (a) 0° < θ ≤ 45° and (b) θ > 45°. At θ = 10°, 20°, 30°, and 45°, which are in group (a), the reduction in θ resulted in better pressure flushing performance. When the submerged vanes are at a slight angle to the flow direction, downflow and rotational currents are generated in front and at the sides of the vanes. Sediments move from the high-pressure zone in front of the vanes to the low-pressure zone behind the vanes. Additionally, rotational flow is created due to the pressure difference on both sides of the vanes. The combination of rotational flow with flow velocity creates a spiral motion downstream of the vanes, which generates transverse shear stress in the bed. Due to the rotational flow downstream of the vanes, sediments are gathered and discharged from the bottom outlet. By reducing the angle of the vanes, the intensity of the vortices around the vanes is increased, which results in the displacement and transfer of more sediment from upstream to downstream of the reservoir. Increasing the inclination angle of the submerged vanes increases the longitude and transverse zones affected by the vanes. The flow hits the front of the vanes and affects the back area less. For this reason, the sediments upstream of the vanes are picked up from the bed, and the sediments downstream of the vanes are transferred with less intensity. Finally, fewer sediment moves with a spiral motion and the dimensions and volume of the sediment flushing cone are reduced.

**Figure 5:**
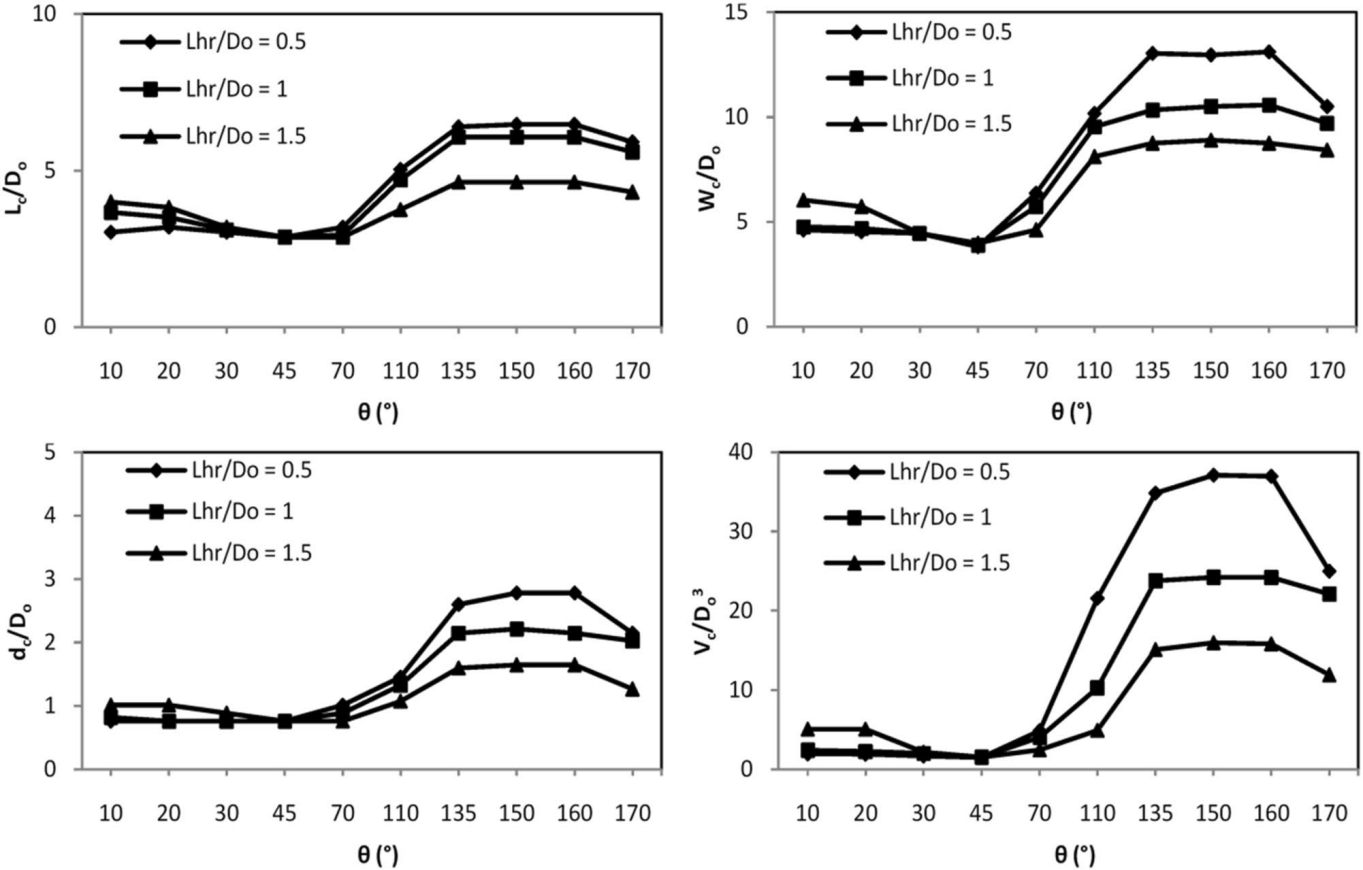
Variations in volumes and dimensions of sediment flushing cones at different skew angles of vanes for $Fr = 2.32$.

Figure 5 shows the variations in the length, width, depth, and volume of the flushing cone at different skew angles of vanes with flow direction and minimum spacing of vanes. This figure shows the increase in flushing cone dimensions with the reduction in θ in group (a) experiments.

To better understand the increased amounts of sediment flushing and the cone dimensions at different skew angles of vanes, Table 2 is presented. It should be noted that due to the large number of experiments, the results of the best experiments (the tests with the maximum dimensions and volume of sediment flushing cone), which in this study is for the maximum discharge (18 l/s or Fr = 2.32) are shown in this table.

**Table 2:** Percent increase in the dimensions and volume of flushing cones compared to the reference test for each discharge.

$Fr$	Angle classification	*θ* (°)	$\frac{{L_{hr} }}{{D_{o} }}$	*∆L*_*c*_ (%)	*∆W*_*c*_ (%)	*∆d*_*c*_ (%)	$\Delta \frac{{V_{c} }}{{D_{o}^{3} }}$(%)
1.6	0° < θ ≤ 45°	10	1.5	155.1	58.333	77.78	578.41
		20	1.5	144.9	50	77.77	575.68
		30	1.5	104.08	16.667	55.56	188.86
		45	1.5	83.673	4.1667	33.34	104.55
1.6	θ > 45°	70	0.5	104.08	66.667	77.78	549.09
		110	0.5	221.43	166.67	155.55	2786.4
		135	0.5	313.27	241.67	333.33	3850
		150	0.5	313.27	239.58	388.88	4865.9
		160	0.5	313.27	218.75	388.9	4695.5
		170	0.5	277.55	175	277.77	2695.5
1.92	0° < θ ≤ 45°	10	1.5	107.31	57.961	60	340.29
		20	1.5	100.25	49.647	60	338.52
		30	1.5	72	16.392	40	87.472
		45	1.5	57.877	3.9216	20	32.75
1.92	θ > 45°	70	0.5	72	66.275	60	321.26
		110	0.5	153.21	166.04	130	1773.3
		135	0.5	216.76	240.86	290	2463.6
		150	0.5	216.76	238.78	340	3122.9
		160	0.5	216.76	218	340	3012.3
		170	0.5	192.05	174.35	240	1714.3
2.32	0° < θ ≤ 45°	10	1.5	104.78	54.92	34.27	238.14
		20	1.5	96.59	46.77	34.27	236.78
		30	1.5	63.83	14.15	17.4	43.98
		45	1.5	47.44	1.92	0.53	1.95
2.32	θ > 45°	70	0.5	63.828	63.077	34.93	223.53
		110	0.5	158.03	160.92	93.96	1338.7
		135	0.5	228.21	234.31	228.9	1868.8
		150	0.5	231.75	232.27	271.06	2375.2
		160	0.5	231.75	235.9	271.06	2366.7
		170	0.5	203.08	169.08	186.73	1293.3

**Figure 6:**
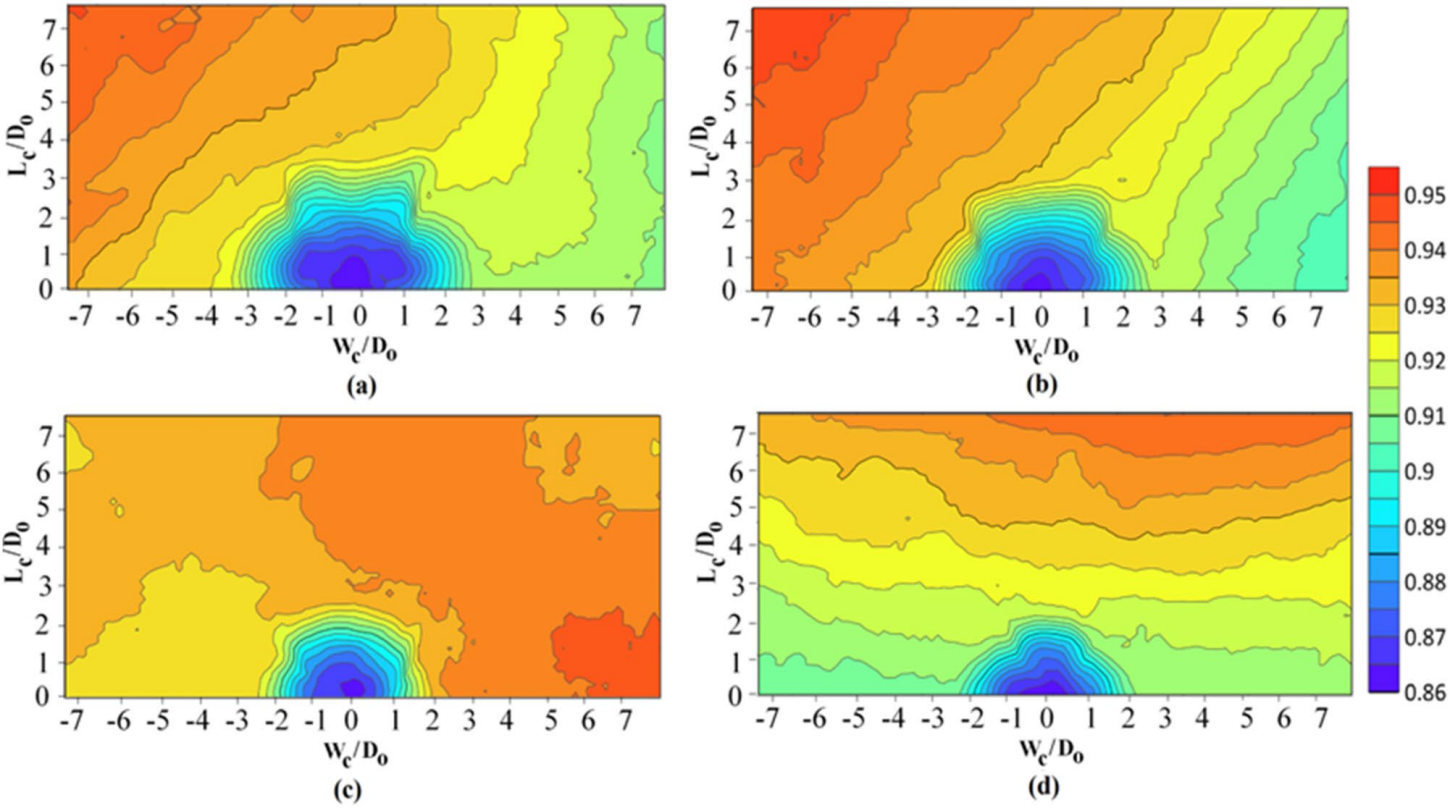
Contour lines of the flushing cones at skew angles of submerged vanes of (**a**) θ = 10°, (**b**) θ = 20°, (**c**) θ = 30°, and (**d**) θ = 45° for $Fr = 2.32{ }$ and $\frac{{{\text{L}}_{{{\text{hr}}}} }}{{{\text{D}}_{{\text{o}}} }} = 1.5$.

It can be observed from this table that at all Froude numbers, in 0° < θ ≤ 45° with increasing angles, the dimensions and volumes of the flushing cones decreased. Additionally, the sediment flushing cones generated by submerged vanes for θ = 10°, 20°, 30°, and 45° are illustrated with contour lines in Fig. 6.

To achieve the maximum size flushing cone, the proper minimum spacing of vanes (${\text{L}}_{{{\text{hr}}}} )$ needs to be considered. It was determined that an increase in $\frac{{{\text{L}}_{{{\text{hr}}}} }}{{{\text{D}}_{{\text{o}}} }}$ from 0.5 to 1.5, the dimensions and volumes of the flushing cones increased for skew angles of θ = 10°, 20°, 30°, and 45°. In this group, the vanes, due to the slight transverse section created between them, have significant interactions, and getting closer to each other will disturb these interactions. At angles of 10°, 20°, 30°, and 45° with increasing minimum spacing of the vanes, the sediments are distributed on a larger surface, which causes the sediment to be transferred to the opposite side of each vane and thus changes the morphological position in the cross-section of the bed. Therefore, the sediment bed rises in one part of the cross-section and falls in the other part. By analyzing Fig. 5, it was determined that at a discharge of 18 L s^-1^ and θ = 10°, the length, width, depth, and volume of the flushing cone increased 1.31, 1.3, 1.33 and 2.6 times, respectively, increasing $\frac{{{\text{L}}_{{{\text{hr}}}} }}{{{\text{D}}_{{\text{o}}} }}$ from 0.5 to 1.5.

#### for skew angles greater than 45°

For skew angles of θ > 45°, scouring was initiated between vanes; thus, flushing cones were produced and expanded upstream of the bottom outlet of the reservoir and transverse sections. It can be concluded from the observations that the scouring was higher downstream than upstream of the vanes. Scour developed several seconds after the start of each experiment. By increasing the skew angle, the upstream side of each vane is entirely exposed to the approaching flow. Likewise, at smaller angles, the effect of the low-pressure side is reduced, but at higher angles, the interference of vortices on both sides of the vanes enhances the scouring rate around the bottom outlet.

**Figure 7:**
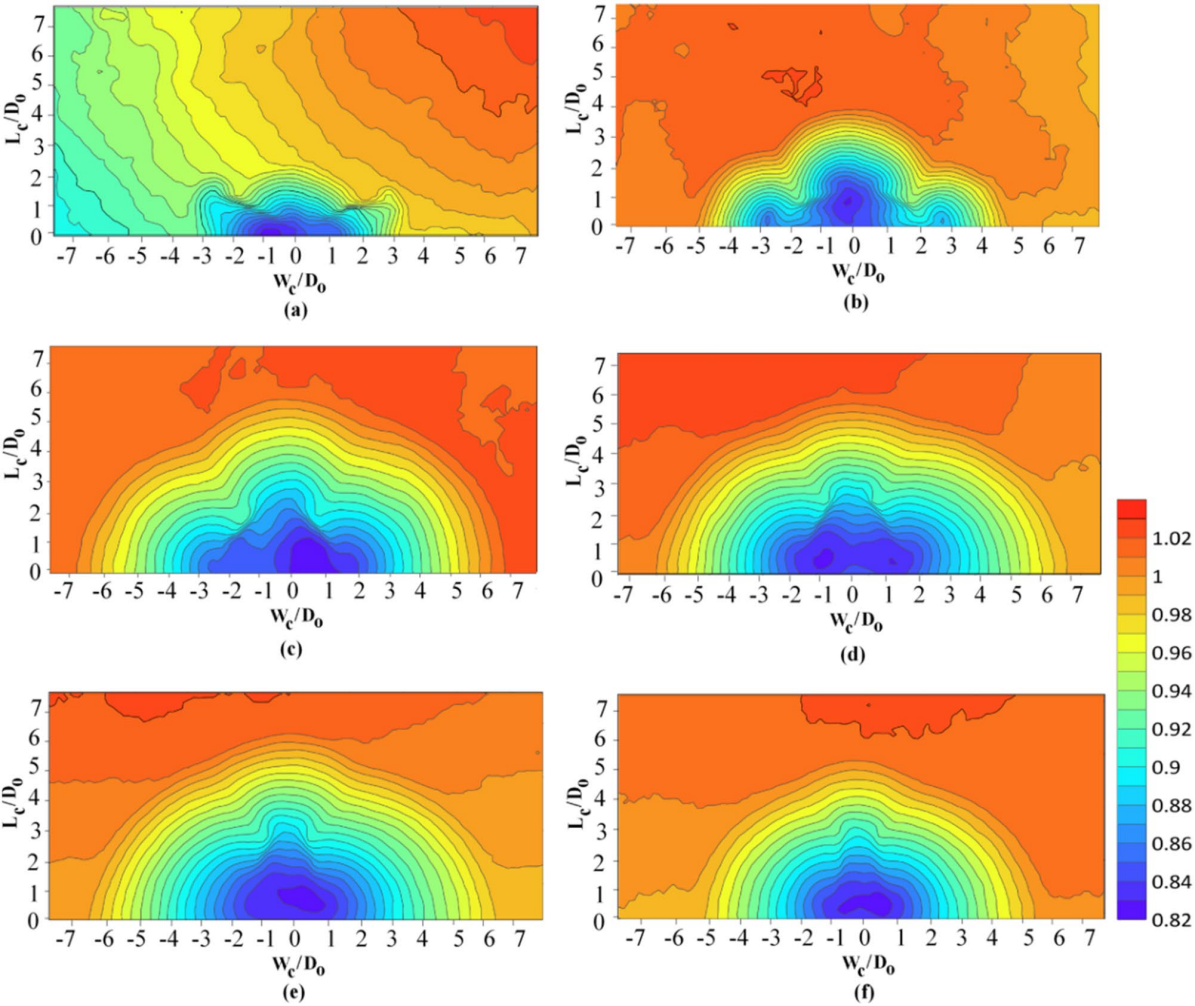
Contour lines of the flushing cones at skew angles of submerged vanes for (**a**) θ = 70°, (**b**) θ = 110°, (**c**) θ = 135°, (**d**) θ = 150°, (**e**) θ = 160°, and (**f**) θ = 170° for $Fr = 2.32$ and $\frac{{L_{hr} }}{{D_{o} }} = 0.5$.

Indeed, the role of the submerged vanes is to create secondary circulation, and changes in the vertical pressure on both sides of the vanes cause rotational flow. Due to the effects on both sides of the vanes at higher vane angles, the combination of this rotation with the velocity in the flow direction causes a helical motion downstream of the vanes, which transfers sediment in a transverse direction^31^. Figure 7 shows an increase in the dimensions and volume of flushing cones from θ = 70° to θ = 160°. It is illustrated in this figure that the evolution of the flushing process was similar for skew angles of θ = 135°, 150°, and 160°. At these angles, due to the more significant interactions of secondary currents with two zones of low pressure and high pressure, scouring originated downstream of the vanes, and flushing cones were generated, expanding to their upstream and downstream sections. The scouring rate increased significantly as soon as all the sediments around the vanes were washed away. The increases in the flushing cone dimensions for θ > 45° are given in Table 2. This table shows that at θ > 45°, an increase in skew angles up to 160° increased the dimensions and volumes of flushing cones, and the maximum variations in the dimensions and volumes of flushing cones in tests with vanes compared to tests without vanes were 135° ≤ θ ≤ 160°.

Figure 7 highlights the influence of the skew angle on the flushing cones with the vanes. The vertical axis represents the length of the flushing cone, and the horizontal axis represents its width. The analysis of Figs. 5 and 7 shows that with increasing vane skew angle, the sediment flushing cones increased for 135° ≤ θ ≤ 160°.

Additionally, Fig. 5 shows that in group (b), a reduction in $L_{hr}$ increases the dimensions of the sediment flushing cones. At θ > 45°, submerged vanes act as individual vortex structures and generate vortices downstream. In fact, in this group, the vanes, due to more created transverse sections than the vanes with θ ≤ 45°, have fewer interactions with each other. Therefore, the reduction of their distance can increase this interplay. When the vanes are placed near each other, vortices interact. These interactions develop interactions between the centrifugal force, the lateral pressure gradient, and circulation, consequently increasing the vortices' strength. The intensity of the vortices increases the strength of the axial flow. Therefore, sediments are picked up with high intensity and carried toward the bottom outlet. Figure 5 shows that at a discharge of 18 L s^-1^, the length, width, depth, and volume of the flushing cone increased 1.4, 1.5, 1.69, and 2.33 times, respectively, with a reduction in $\frac{{{\text{L}}_{{{\text{hr}}}} }}{{{\text{D}}_{{\text{o}}} }}$ from 1.5 to 0.5.

### Effects of different skew angles of vanes on longitudinal and transverse sections of the flushing cone

**Figure 8:**
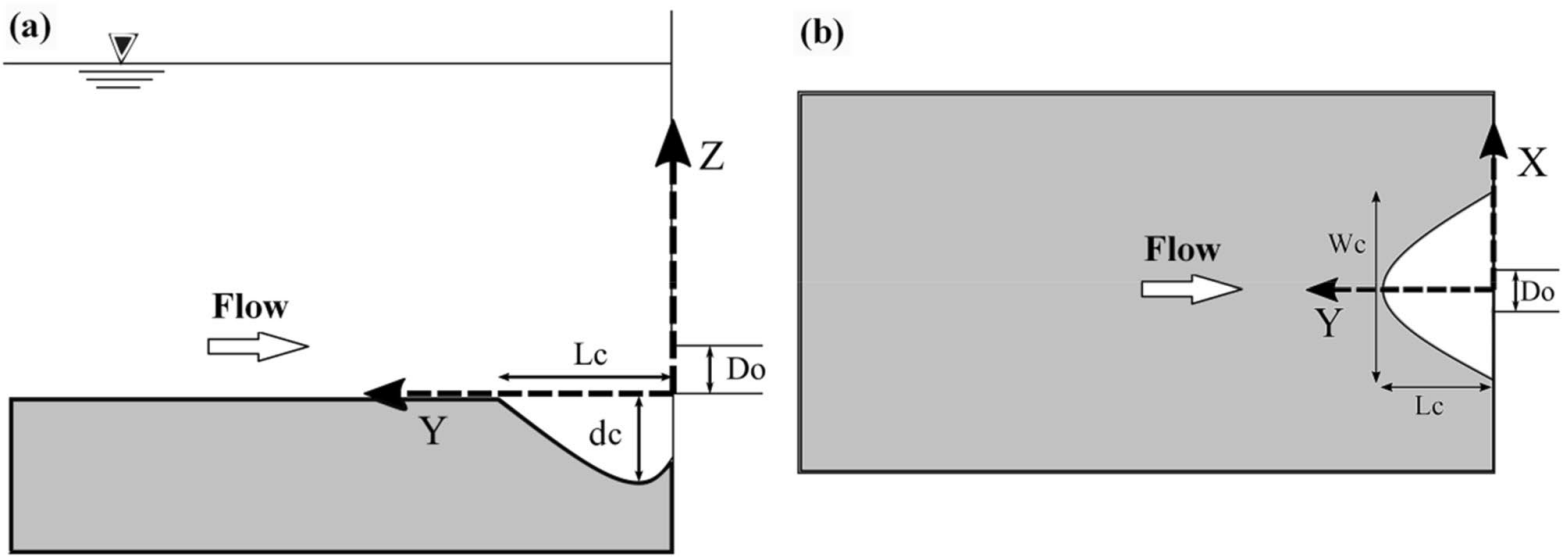
(**a**) Side view, (**b**) plan view of the coordinate system of the experiments.

**Figure 9:**
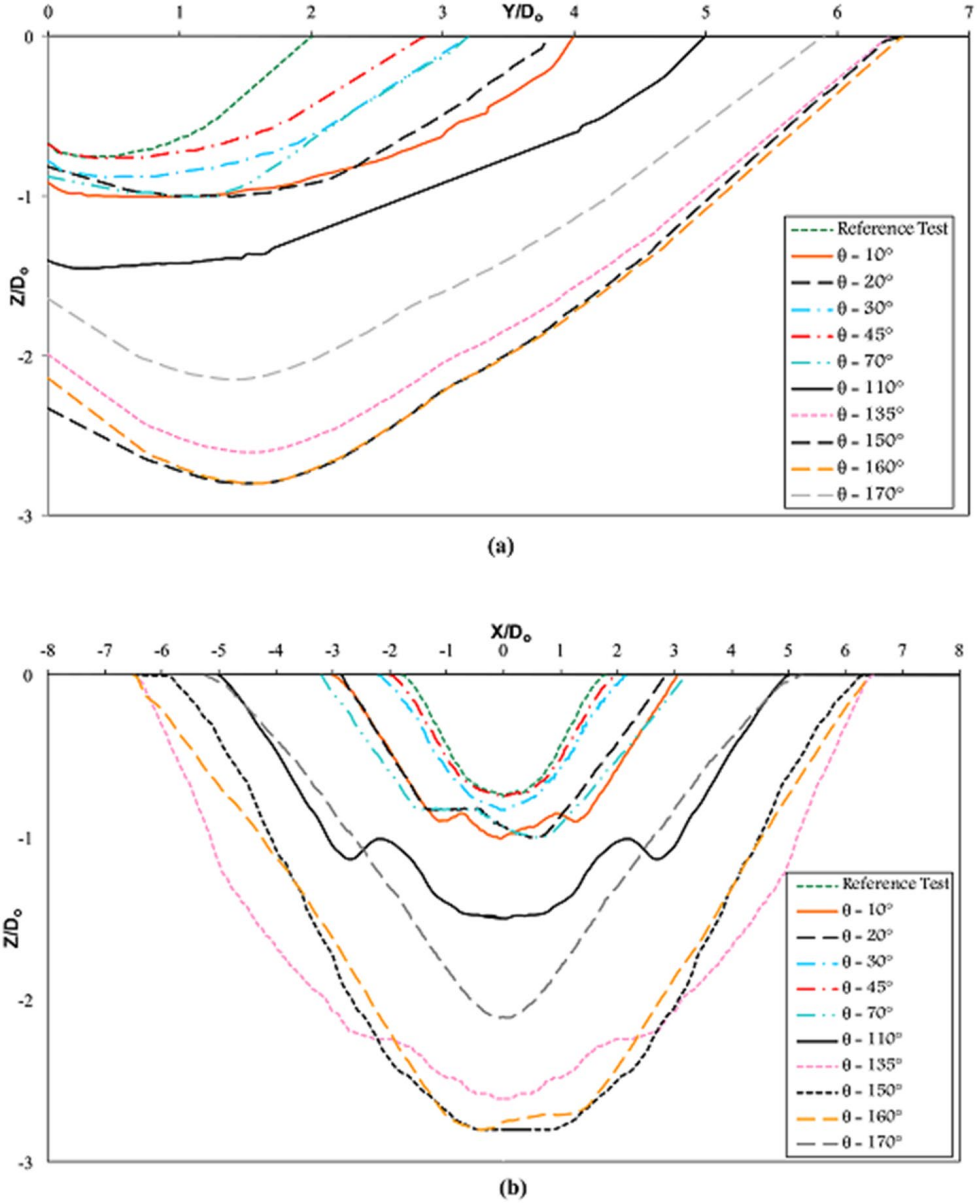
Nondimensional (**a**) longitudinal and (**b**) transverse profiles of flushing cones at different skew angles of submerged vanes for $Fr = 2.32$ and $\frac{{L_{hr} }}{{D_{o} }} = 1.5$ in θ ≤ 45° and $\frac{{L_{hr} }}{{D_{o} }} =$ 0.5 in θ > 45°.

To observe the performance of vanes with different angles on sediment flushing cone dimensions, according to the defined coordinate system shown in Fig. 8, longitudinal and transverse sections are drawn (Fig. 9). In this figure, the X, Y, and Z-axes are standardized with the bottom outlet diameter (${\text{D}}_{{\text{o}}}$). By analyzing Fig. 9, it can be concluded that there are significant differences in the scour cone size for 135° ≤ θ ≤ 160°in both longitudinal and transverse directions compared to the reference test and test with different angles. This can be explained by the smaller angles having a narrower cross-section and, thus, the minor influence in the transverse direction.

The bed slopes of the scour cones have also been studied extensively, and several comparisons have been made between the slope angles of the longitudinal and transverse axes. The consensus is that for noncohesive sediment, the scour cone slopes would be approximately the same as the submerged angle of repose of the deposits. Many researchers have noted that the transverse bed slope is slightly steeper than the longitudinal bed slope^41,52,53^.

Each test's longitudinal and transverse scour cone slopes were determined from the deepest point in the scour profile up to its upstream end. The average longitudinal and transverse slopes in tests with different skew angles of vanes were 29° and 33.8°, respectively. It was concluded that the transverse slope is steeper than the longitudinal slope, consistent with previous studies.

### Proposed equation of flushing cone volume

To estimate the volume of the flushing cone with the independent parameters in Eq. (3), an equation for the volume of the scour cone was developed based on a statistical analysis of the results. The equation was verified with experimental measurements in this study's flushing events. The data for generating this equation was divided into two groups, one is about 80% for training the equation and the rest for testing. Equation (4) for the scour volume was developed and is presented below. Notably, this equation was in the ranges of 135° ≤ θ ≤ 160°, 1.6 ≤ ${\text{Fr}}$ ≤ 2.32, and 0.5 ≤ $\frac{{L_{hr} }}{{D_{o} }}$ ≤ 1.5.

4 $$ \frac{{V_{c} }}{{D_{o}^{3} }} = 5.18328 \left( {Fr} \right)^{1.54372} \left( {\frac{{L_{hr} }}{{D_{o} }}} \right)^{ - 0.65341 } \left( {\sin \theta } \right)^{ - 0.189312} $$

**Figure 10:**
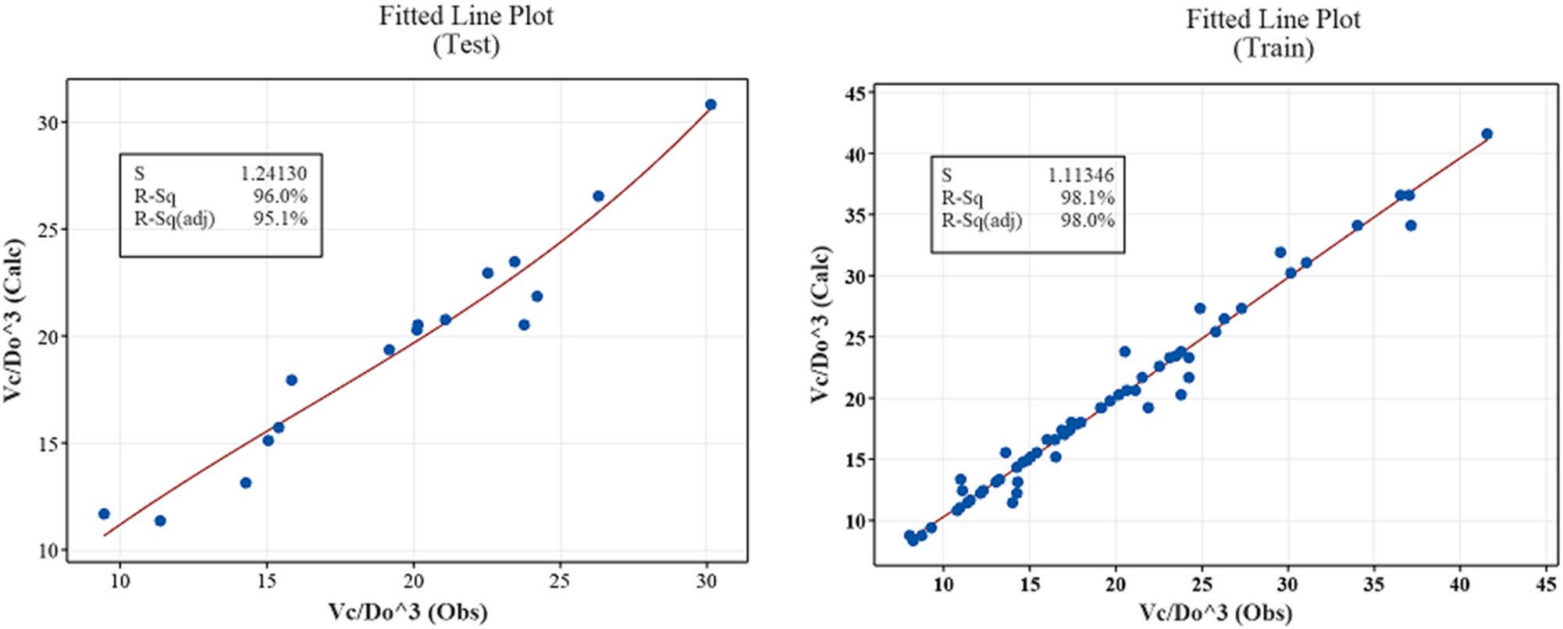
Observed versus predicted cone volume (for test and train data).

Distinguishing between the observed and predicted values was possible by analyzing the statistical parameter of the R-squared value (R^2^). The results show that the proposed equation has a high value for the coefficient of determination (R^2^), which was very good considering the variables tested for different independent parameters, and the predicted and measured data correlated well. The computed and measured scour volume variations using the proposed equations are presented in Fig. 10. It can be concluded from this figure that there is good agreement between the observed and calculated values.

## Conclusions

In this study, the performance of submerged vanes aligned with different skew angles and different minimum spacing was evaluated to increase the performance of pressure flushing. The tests were conducted with and without vanes at different discharges. The results revealed that the use of submerged vanes increased the performance of pressure flushing. An increase in outflow discharge significantly affected the development of a more oversized flushing cone. As the discharge increased 1.44 times, the volume of the flushing cone increased almost four times. Analyzing different skew angles of vanes showed that for θ ≤ 45°, decreasing the skew angle (θ) and increasing the minimum spacing of vanes (L_hr_) increased the volume and dimensions of the flushing cone. Consequently, increasing the skew angle decreased the performance of the vanes for θ ≤ 45°. For vanes with θ > 45°, a reduction in (L_hr_) increased the pressure flushing performance, and the flushing cone geometry developed with increasing skew angle reached the maximum value at θ = 160° and then decreased. According to the analysis of the results, the maximum volume and dimensions of flushing cones appeared at 135° ≤ θ ≤ 160°, where the volume of the flushing cone in the discharge of 18 L s^-1^ increased 25 times compared to that during the test without submerged vanes. Furthermore, nonlinear regression analysis developed a nondimensional equation for the scour cone volume. The presented equation has a high correlation coefficient concerning experimental data and predicts the scour cone geometry with acceptable accuracy. The results of this study can help natural dams maintain intakes free from sediment. For extra research, the flow pattern of using submerged vanes upstream of the bottom outlets in pressure flushing is suggested to be investigated. Additionally, the effect of the vortices due to the submerged vanes on vortices generated by the bottom outlet should be studied to answer this question: are the vortices near the bottom outlet enhanced by the presence of the vanes or do the vane-induced vortices dominate the sediment removal process? Resolving these challenges can expand this study and complete the results yet to be filled in the knowledge about submerged vanes in front of bottom outlets.

## Data Availability

The datasets used and analyzed during the current study are available from the corresponding author upon reasonable request.

## List of symbols

$L_{hr}$: The minimum spacing of submerged vanes (cm)
$L_{v}$: Distance of submerged vanes from the bottom outlet (cm)
$H_{sv}$: Height of submerged vanes above sediment bed (cm)
$L_{sv}$: Length of submerged vanes (cm)
${\uptheta }$: Inclination angle of submerged vanes on the flow direction (°)
L_c_: Length of sediment flushing cone (cm)
W_c_: Width of sediment flushing cone (cm)
d_c_: Depth of sediment flushing cone (cm)
V_c_: Volume of sediment flushing cone (m^3^)
H_w_: Water head (cm)
H_s_: Sediment level (cm)
Do: Diameter of the bottom outlet (mm)
d50: Median grain size (mm)
Fr: Froude number (–)
Re: Reynolds number (–)
*g*: Gravity acceleration (m s^−^^2^)
*G*_*s*_: Sediment specific gravity (ton m^−^^3^)
Re: Reynolds number (–)
$\mu$: Fluid viscosity (Pa s)
$\rho_{w}$: Fluid density (kg m^−^^3^)
$\rho_{s}$: Sediment density (kg m^−^^3^)
